# Orbital Myiasis: Due to Invasion of Larvae of Flesh Fly (*Wohlfahrtia magnifica*) in a Child; Rare Presentation

**DOI:** 10.1155/2012/371498

**Published:** 2012-02-01

**Authors:** R. P. Maurya, Deepak Mishra, Prashant Bhushan, V. P. Singh, M. K. Singh

**Affiliations:** ^1^Department of Ophthalmology, Institute of Medical Sciences, Banaras Hindu University, Varanasi 221005, India; ^2^Regional Institute of Ophthalmology, IGIMS, Patna, Bihar 800014, India

## Abstract

*Wohlfahrtia magnifica* larvae cause myiasis in mammals, mainly in sheep and rarely in human. In human *it* may infest the ear, eye, mouth or nose, damaging living tissues. We report a case of ocular myiasis in 1.5 years old child belonging to urban slum after history of minor injury on left upper lid due to fall from bed. The purpose of reporting this case is to highlight the ocular association of *W. magnifica*.

## 1. Introduction

The term myiasis is derived from the Greek word “Myia” meaning fly. Myiasis is defined as the infestation of living tissue of human and other vertebrate animals by the eggs or larvae of flies of the Orthopoda order Diptera. Although myiasis in human beings is generally rare, it is sometimes seen in tropical and subtropical countries having overcrowded condition, poor environmental sanitation and personal hygiene [1–3]. Worldwide the most common flies that cause human infestation are *Dermatobia hominis* (human boat fly) and *Cordylobia anthropophaga* (tumbu fly). Ophthalmomyiasis involves the eye, orbit, and periorbital tissues [4]. Human ophthalmomyiasis was first reported by Keyt in 1900 and later on by Elliot from India in 1910 [5]. 

 The parasite most commonly affecting the eye and orbit is the larva of *Hypoderma bovis* (hornet fly) which infests cattle and rarely by *Wohlfahrtia magnifica* (flesh fly) [5, 6]. Orbital involvement occurs in approximately 5% of all cases of myiasis [4].

## 2. Case Report

A one-and-a-half-year-old boy, son of a sweepress, belonging to urban slum, visited us with history of minor injury on left upper lid due to fall from bed. He presented to us six days after the injury with chief complaints of swelling, itching, and blood-stained foul-smelling discharge from the wound. Parents also noticed small whitish yellow worm-like structures crawling over the wound. 

 On examination of the left eye there was solid oedema of both lids and large ulcer of about 3 cm × 1 cm between left eye brow and left upper lid extending up to medial canthus. Ulcer margins were inflamed and indurated. Freely moving maggots were observed through an opening of the ulcer. There was scanty blood-stained foul-smelling discharge seen from the wound. Detailed ocular examination was not possible because retraction of lid was very difficult due to solid oedema. However marked conjunctival chemosis were seen.

**Figure 1:**
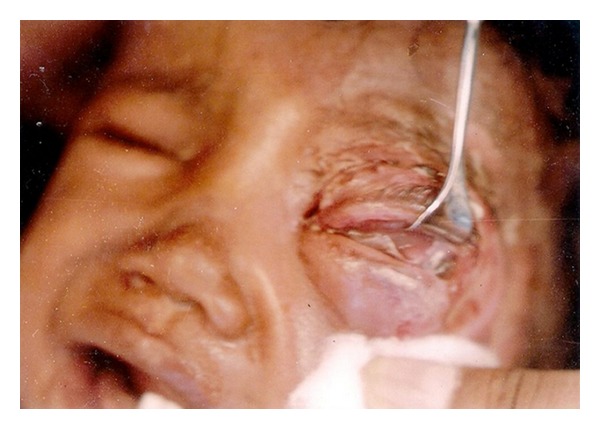
Pre treatment photograph of *W. magnifica*.

**Figure 2:**
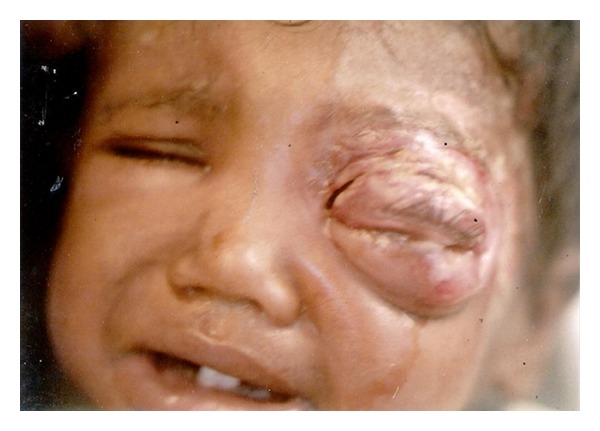
Pre treatment photograph showing chemosis and ulcer.

**Figure 3:**
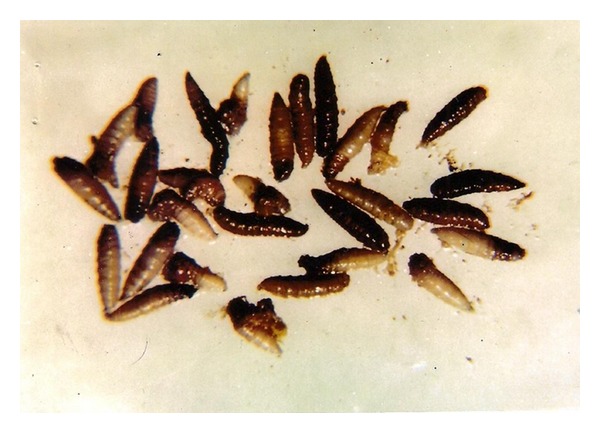
*W. magnifica* maggot removed from eye lid after surgery.

**Figure 4:**
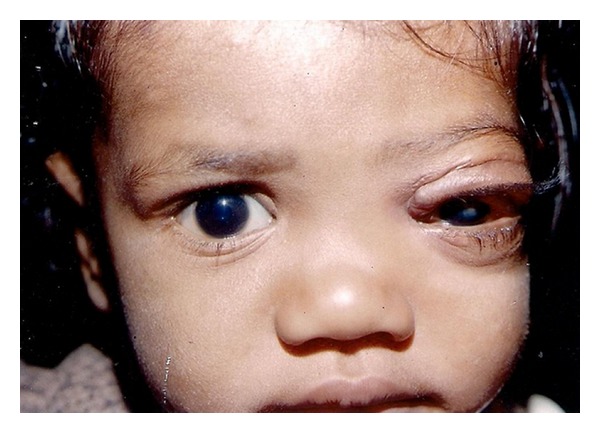
Post treatment photograph of same child.

 Mechanical removal of the maggots was done with the help of forceps after immobilizing the larvae by applying 4% xylocaine and mixture of chloroform plus turpentine oil packing. Regular dressing and removal of maggots was done for five days. Routine topical and systemic antibiotics along with anti-inflammatories were administered. The ulcer healed within two weeks. Thirty-five larvae were removed and preserved in diluted formalin and were examined later on by an entomologist and identified as larvae of flesh flies (Figures 1–4).

## 3. Discussion

Ophthalmomyiasis is a rare form of eye morbidity. Patients of ophthalmomyiasis may present with clinical signs and symptoms of irritation and inflammation to total destruction of the orbit [2]. Ophthalmomyiasis is classified into external and internal (or orbital) according to the site of the larval infestation. In external ophthalmomyiasis superficial periocular tissues get infested, and it can be subclassified into palpebral and conjunctival myiasis. In internal ophthalmomyiasis larvae penetrates the conjunctiva and sclera and migrates into subretinal space. Orbital myiasis is marked by large number of larvae invading and destroying the tissue contents with complications ranging from minor ocular irritation to complete blindness [7, 8].

 Normal healthy individuals are unlikely to suffer from myiasis [2]. The main predisposing factors for the larval infestation in our patient were probably illiteracy, lack of self hygiene, and overcrowding. Flies often get attracted to the wounds due to foul smelling arising from the ulcer. Their eggs can be either directly deposited in and around wounds or indirectly transferred to it by the patients' own fingers while scratching. The key step in management of less extensive ophthalmomyiasis is mechanical removal of maggots with forceps after suffocating them with use of various chemical substances like turpentine oil with or without chloroform which blocks the spiracles of larvae [5]. Exenteration may be needed to prevent intracranial extension of tissue destruction in case of massive orbital myiasis.

## References

[1] Ratnakar KS, Lakshminarayan CA, Ramachandraiah U (1974). Ocular myiasis due to Oestridae. *Indian Journal of Ophthalmology*.

[2] Duke- Elder S (1958). *System of Ophthalmology*.

[3] Kersten RC, Shoukrey NM, Tabbara KF (1986). Orbital myiasis. *Ophthalmology*.

[4] Burns DA (1094). Diseases caused by Arthropods and other Noxious animals. *Rooks Textbook of Dermatology*.

[5] Agarwal DC, Singh B (1990). Orbital myiasis—a case report. *Indian Journal of Ophthalmology*.

[6] Mathur SP, Makhija JM (1967). Invasion of the orbit by maggots. *British Journal of Ophthalmology*.

[7] Weinand FS, Bauer C (2001). Ophthalmomyiasis externa acquired in Germany: case report and review of the literature. *Ophthalmologica*.

[8] Sachdev MS, Roop HK, Jain AK, Arora R, Dada VK (1990). Destructive Ocular Myiasis in non-compromised host. *Indian Journal of Ophthalmology*.

